# Psychosis Triggered by Intensive Meditation: A Case Report and Review of Risk Factors

**DOI:** 10.1192/bjo.2025.10726

**Published:** 2025-06-20

**Authors:** Mariana Galvao de Oliveira

**Affiliations:** South London and Maudsley NHS Foundation Trust, London, United Kingdom

## Abstract

**Aims:** Meditation is widely regarded as a beneficial practice for mental well-being, but intensive forms, such as those practiced during retreats, can pose risks. In vulnerable individuals, prolonged meditation may trigger psychosis. This case explores a psychotic episode in a previously healthy individual during an intensive meditation retreat, with a focus on clinical presentation, management, and implications for practice.

**Methods:** Case report.

Patient overview: Demographics: Female, 31 years old, with no ongoing mental health treatment. Psychiatric history: Previous drug-induced psychosis 6 years ago, resolved without recurrence. Substance use: Denied drug use since the prior episode. Admission toxicology screening (urine drug screen) was negative.

Retreat context: Attended a 7-day meditation retreat involving intensive mindfulness practices, minimal social interaction, and prolonged sitting meditations. Psychotic symptoms began after 3 days, prompting early withdrawal from the retreat.

Clinical presentation: Visual hallucinations: Reported seeing people’s faces transform into demonic appearances. Auditory hallucinations: Hearing voices reinforcing delusions. Persecutory delusions: Believed she and her family were in grave danger, and that her death was the only way to save them. Behavioural changes: Heightened distress and withdrawal from the retreat.

Management and outcome: Admitted to the psychiatric unit. Started on olanzapine 5 mg daily. Rapid symptom resolution within 6 days. Discharged with no residual psychotic symptoms.

Literature review: Intense meditation practices, especially during retreats, can lead to adverse psychological effects, including psychosis, depersonalisation, and emotional dysregulation. Risk factors identified in literature:

Pre-existing vulnerability (e.g., history of psychosis or trauma).

Retreat conditions (e.g., fasting, sleep deprivation, and isolation).

Lack of individualised guidance or screening.

Meditation-induced psychosis has been noted to present with symptoms such as hallucinations, paranoia, and altered states of consciousness. Recovery is typically rapid with antipsychotic treatment.

**Results:** Mechanisms: Prolonged meditation may disrupt normal cognitive and emotional regulation, leading to altered reality testing. Psychotic symptoms could result from sensory deprivation, emotional overload, or resurfacing of unresolved trauma.

Case-specific insights: While the patient had a history of drug-induced psychosis, her 6-year symptom-free period and negative toxicology suggest that meditation-induced stress was the primary trigger. The rapid response to low-dose olanzapine highlights the transient nature of the condition.

Implications for practice: Pre-retreat mental health screenings are crucial to identify vulnerable individuals. Retreats should offer tailored practices and provide professional mental health support. Awareness among clinicians is necessary to distinguish between culturally induced altered states and pathological psychosis.

**Conclusion:** This case underscores the potential for intensive meditation to induce psychosis, even in individuals without active mental illness. Clinicians and meditation facilitators must collaborate to mitigate risks, particularly for individuals with prior psychiatric vulnerabilities.

